# Leptomeningeal CNS Relapse in Multiple Myeloma Post Autologous Stem Cell Transplant– the Mystery of Cytogenetic Clones in CSF

**DOI:** 10.1007/s12288-025-02054-3

**Published:** 2025-06-04

**Authors:** Sumeet Mirgh, Avinash Gupta, Malini Garg, Anil Yadav, Shreya Shukla, Anil Singh, Anant Gokarn

**Affiliations:** 1 Homi Bhabha National Institute (HBNI), Mumbai, Maharashtra India; 2 Homi Bhabha Cancer Hospital (HBCH), Varanasi, Uttar Pradesh India; 3 Department of Medical Oncology, Adult Hematolymphoid and BMT Unit, Tata Memorial Centre, ACTREC, Kharghar, Navi Mumbai, Maharashtra 410210 India

**Keywords:** Multiple Myeloma, Leptomeningeal CNS relapse, Relapse, Intrathecal dexamethasone, FISH in CSF, Bendamustine-Pomalidomide-Dexamethasone

A 51-year-old diabetic male, presented with shoulder pain in June 2022. Serological tests revealed monoclonal IgA-kappa of 2.5gm/dl. Bone marrow (BM) revealed 15% plasma cells (PC) with hyperdiploid trisomic phenotype (trisomy-5,9,15) and 1q21 gain. Baseline PET-CT revealed multiple lytic lesions with soft-tissue component and supra-diaphragmatic lymphadenopathy (largest lymph-node right supraclavicular lymph-node 3.1 × 2.7 cm). He did not have constitutional symptoms, prior personal/family history of tuberculosis, and the lymph-nodes were non-necrotic without any contrast enhancement. Attributing his lymphadenopathy to myeloma, he was started on VRd. After 6-cycles VRd, he achieved a complete response, and underwent autologous stem-cell transplant (ASCT) with Melphalan (200 mg/m2). Prior to ASCT, he was in CR (both BM and PET-CT), but BM flow-cytometry MRD-positive 0.3%. On day + 100, just prior to start of lenalidomide maintenance, he presented with 5-day history of intractable headache without any focal neurodeficit. MRI did not reveal any parenchymal abnormality. CSF-cytology revealed 125 cells/mm^3^ (all PCs), with flow-cytometric evidence of abnormal clonal-PCs (Fig. 1). Intriguingly, his CSF-FISH showed same cytogenetic PC-clone with trisomic phenotype, although 1q was negative (Fig. 1). Since serological tests did not show any biochemical relapse, BM examination was not done. Hence, he was labelled as an isolated leptomeningeal relapse post-ASCT.

**Fig. 1:**
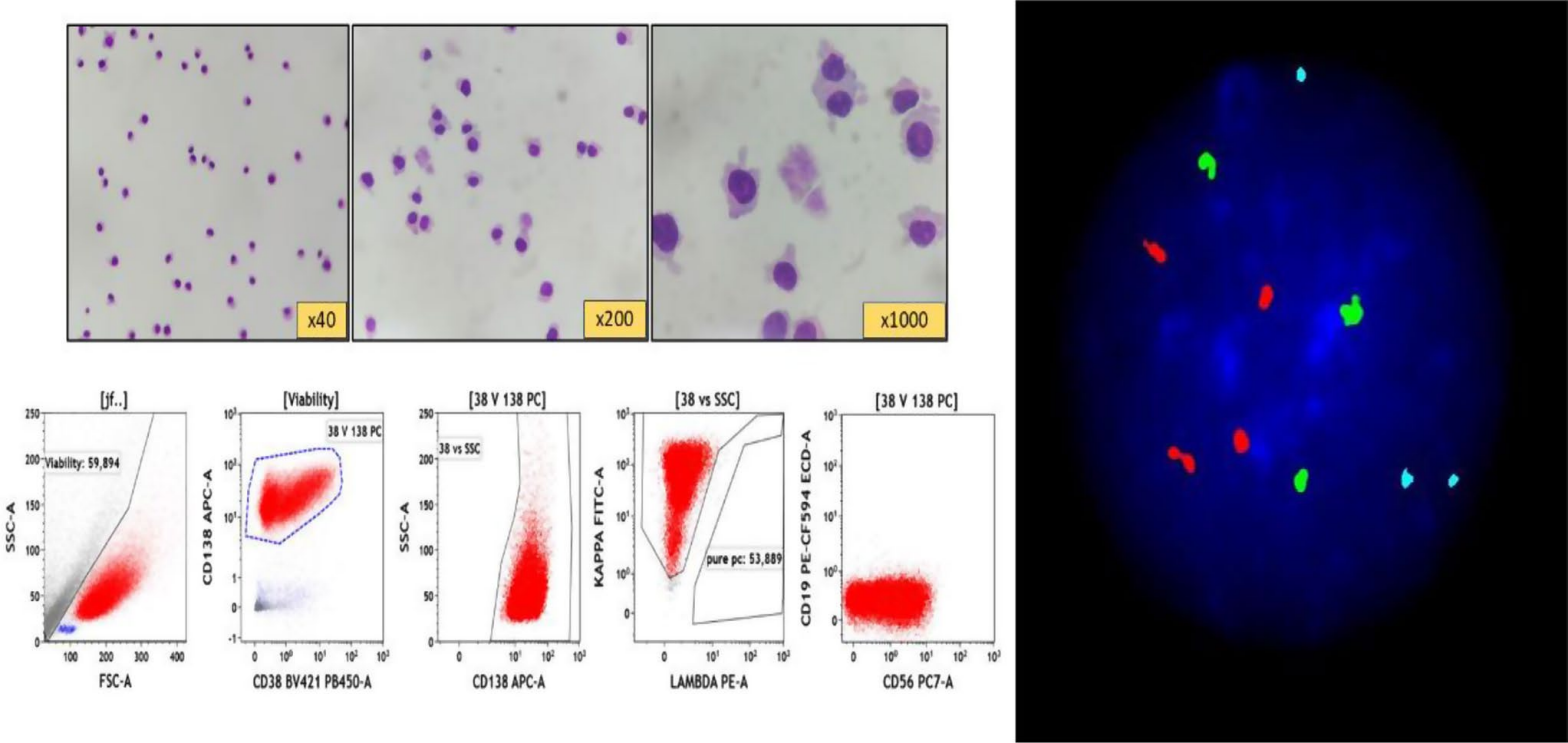
*(Upper left panel)* CSF microscopy: Cytocentrifuged smear showing plasma cells with occasional binucleated form; *(Lower left panel)* Multiparametric flowcytometry (MFC) of CSF revealed abnormal clonal Plasma cells (Red population) gated at CD38 vs. CD138. These abnormal clonal plasma cell express variable CD38, moderate CD138, variable CD56, kappa-light chain restriction and negative for CD19 expression; *(Right panel)* - Fluorescent in situ hybridization (FISH) panel showed trisomic phenotype [three green (trisomy of *chromosome 5*), three aqua signal (trisomy 9) in 80% cells, four orange (tetrasomy chr 15)], and negative for IgH breakapart, 1q gain/amplification and TP53 deletion on CSF samples. Only fifty cells were analysed in each marker because of CSF sample

He was treated with Bendamustine [1] (70 mg/m2 days-1,2,14,15 in 28-day cycle) (70 mg/m2 dose in-view-of recent ASCT contemplating a compromised BM reserve) + Pomalidomide [2] (4 mg PO Day1-21/28) + 40 mg weekly dexamethasone (BPd), along with triple intra-thecal (Hydrocortisone + Methotrexate + Cytarabine) therapy (TIT). He had significant symptomatic relief after first TIT. After 3-cycles BPd, his bendamustine had to be stopped in view of persistent ≥ grade-3 cytopenias (inspite of intermittent G-CSF), and pneumonia needing prolonged hospitalization. Pomalidomide-dexamethasone were continued. He received TIT (twice-a-week for 3 months, followed by once-a-week for 3 months), although his CSF-cytology continued to show PCs. After 6 months, dexamethasone (4 mg diluted in 5 ml 0.9%NS) [3] was substituted for hydrocortisone in his TIT (modified TIT). After two doses of dexamethasone-based modified TIT, his CSF became negative, and he was continued on that once-a-week. Eight months after modified TIT, he had an episode of seizures. Imaging (Fig. 2) showed new-onset parenchymal lesions. He underwent palliative radiotherapy for his parenchymal lesions, without any improvement. He succumbed to his illness 14-months after diagnosis of CNS relapse. Daratumumab was not financially feasible, and marizomib [4] is not available in India. Moreover, daratumumab has limited role in extra-medullary disease [5], with poor CNS penetration [6]. Our patient had variable CD56 expression in CSF; which had a dim expression in the diagnostic marrow. CD56 is pivotal in holding plasma-cells and keeping them anchored to the bone marrow stroma, thereby limiting their extramedullary spread [7]. Whether dim expression of CD56 was responsible in its CNS spread at relapse is debatable. Our case is unique, as CNS myeloma is a rare entity (< 1% of all myelomas) [2], there was an early isolated leptomeningeal relapse post-ASCT, we could demonstrate the same cytogenetic PC-clone in CSF, with a 14-month survival and a possible benefit with modified TIT. Loss of 1q in CSF-FISH was an intriguing finding. To our knowledge, this is the first report to show loss of 1q clone in CSF, thereby highlighting the role of FISH in CSF for generating new knowledge. Whether his baseline lymphadenopathy, contiguous soft-tissue component with bone lytic lesions or IgA-isotype made him more vulnerable to a CNS relapse is unknown.

**Fig. 2:**
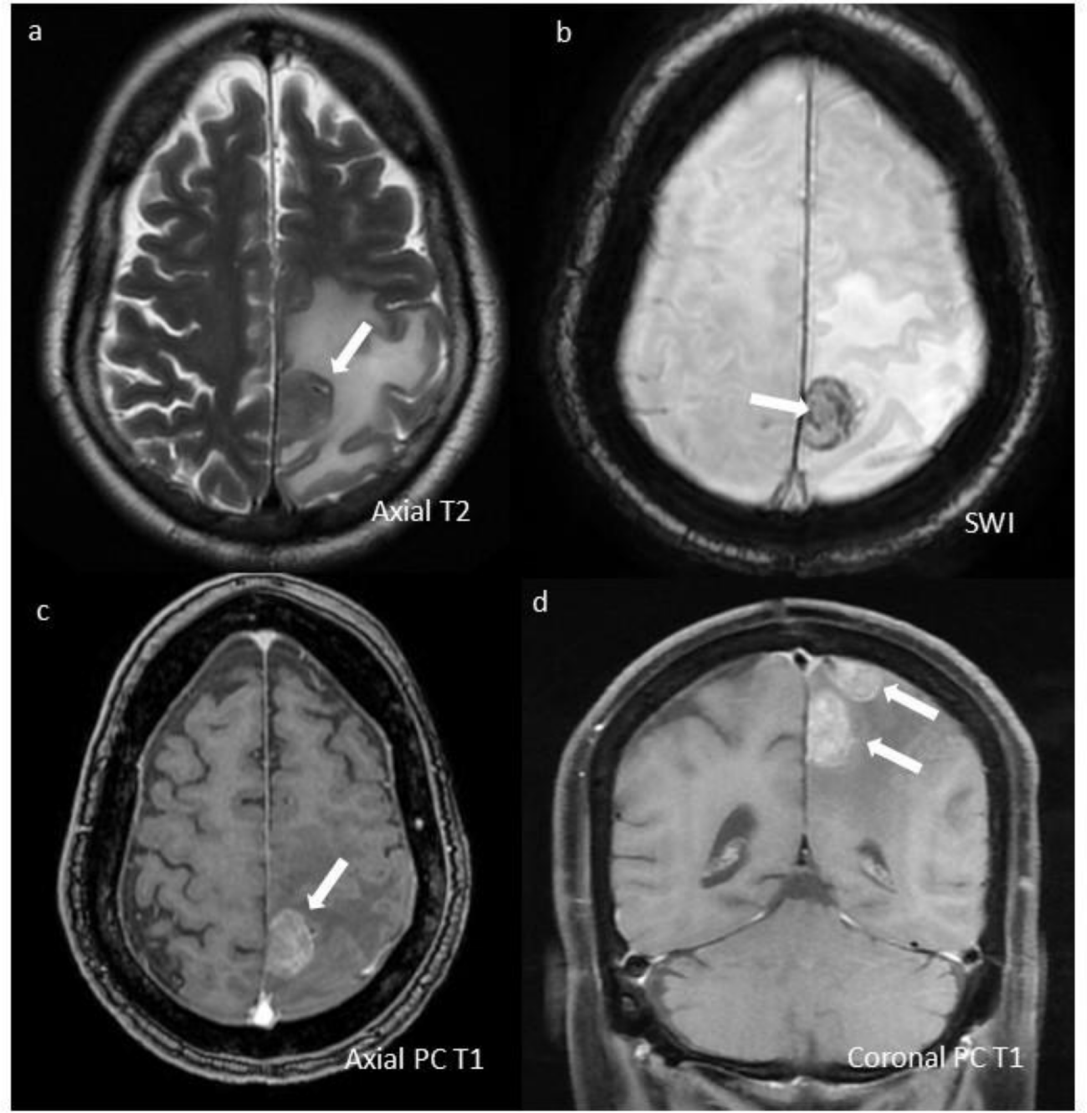
Contrast-enhanced MRI images show well-defined, extra-axial dural-based lesions (marked with white arrows) in left high parietal para-falcine region. (**a**) Lesion has intermediate T2 signal with significant surrounding T2 hyperintense vasogenic edema. (**b**) It shows blooming on SWI suggesting intralesional hemorrhage. (**c**,**d**) Two similar morphology lesions showing heterogeneous moderate enhancement are seen on post contrast T1w images

## Data Availability

Available from corresponding author on request.
